# Exploring Cationic Substitutions in the Solid Electrolyte NaAlCl_4_ with Density Functional Theory

**DOI:** 10.1021/acs.jpcc.4c05559

**Published:** 2024-11-15

**Authors:** Michael Häfner, Matteo Bianchini

**Affiliations:** † Faculty of Biology, Chemistry and Earth Sciences, Universität Bayreuth, Universitätsstrasse 30, 95447 Bayreuth, Germany; ‡ Bavarian Center for Battery Technology (BayBatt), Universität Bayreuth, Weiherstrasse 26, 95448 Bayreuth, Germany

## Abstract

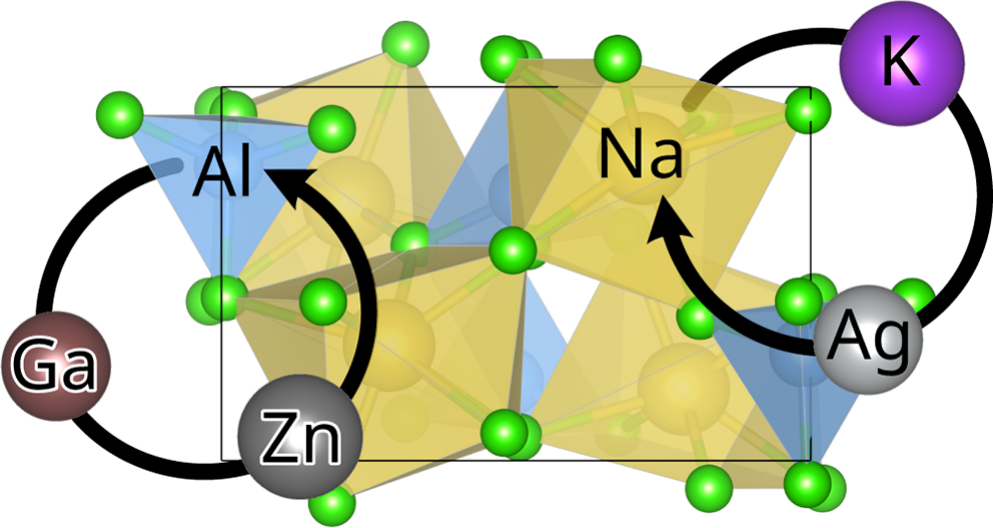

NaAlCl_4_ is an established solid electrolyte in high-temperature Na-based battery systems, but its ionic conductivity is not sufficiently high for room-temperature applications. We employ density functional theory and thermodynamic corrections to evaluate the efficacy of various elements for substitution, utilizing on-the-fly machine-learned potentials to accelerate the required phonon calculations by 1 order of magnitude at a minor error of −0.7 ± 1.0 meV/atom. All investigated isovalent substitutions are favorable within 4 meV/atom, with potassium and silver as substitutes for sodium and gallium as a substitute for aluminum. The most promising aliovalent substitution was identified for Zn on the tieline between NaAlCl_4_ and Na_2_ZnCl_4_. The structure of latter, with aluminum ions replacing zinc, yields a structure with separate layers for the differently charged cations and vacancies for potential Na conduction. Our investigation may pave the way for more reliable discovery of new Na conductors by inclusion of thermodynamic properties.

## Introduction

Due to the growing prevalence of renewable but more volatile energy sources like wind and solar,^1^ the requirement for energy storage media with high capacity and low cost is greater than ever. One such storage medium is the lithium-ion battery, which is widespread as it provides enough capacity per weight and volume to effectively power everything from mobile phones to cars.^2,3^ However, lithium-ion batteries are inherently dependent on lithium for their production, which, while not particularly rare in the Earth’s crust (≈20–70 ppm),^4^ still constitutes a significant source of issues since only few countries like Chile, Australia, and China currently dominate the production.^5^ Moreover, the price underwent significant fluctuations in the past (e.g., rise of 1500% for Li_2_CO_3_ between 2020 and 2022),^6^ and the mining and refining process causes significant environmental and human rights issues.^7^ Hence, a new field of battery technology has arisen that focuses on using sodium instead of lithium,^8,9^ which is abundant and readily available in rock salt deposits and seawater.^10^ Another drawback of the standard lithium ion battery is its liquid or gel electrolyte, which creates issues regarding flammability and the accessible voltage window.^11−13^ Solid electrolytes are a potential way of addressing these problems,^14−21^ and one such candidate for sodium-based solid-state batteries, NaAlCl_4_, is the focus of this work.

Due to this material’s relatively low melting point of 426 K,^22^ it has already found use as a liquid electrolyte in synthesis^23^ and high temperature sodium–nickel chloride batteries (also called ZEBRA batteries).^24^ Its electronic conductivity is very low at room temperature (1.2 × 10^–10^ S cm^–1^), and it is stable up to a voltage of 4 V against Na/Na^+^, which allowed the demonstration of its use as catholyte in Na-based solid state batteries.^25^ However, the ionic conductivity of NaAlCl_4_ is about 3.9 × 10^–6^ S cm^–1^ at room temperature (RT), which is acceptable, yet 3 orders of magnitude below superconducting solid-state electrolytes such as NASICON, sulfides, and antiperovskites.^21^

One possible method to enhance the ionic conductivity is the introduction of defects or dopants, the latter of which is the focus of this work. Substitutional elements can, for example, create vacancies that are paramount for ionic conductivity (especially if the migration is vacancy-mediated); can modify the crystal structure, thereby reducing the activation barriers for diffusion; or can increase the density of charge carriers, hence directly increasing conductivity, as proven for various lithium- and sodium-based solid electrolytes.^26−29^

Employing density functional theory (DFT) on the generalized gradient approximation (GGA) level, we investigate the elements Ag, K, Mg, Ca, Sr, Ba, Sc, Y, La, Zn, and Ga regarding their suitability as substitutes for either Na or Al in NaAlCl_4_. These choices comprise some that are interesting for applications in batteries and some that are explored at a more fundamental level. In the first section, we assess the general viability of various substituting ions for NaAlCl_4_. Second, we examine how the calculation of vibrational thermodynamics can be accelerated by utilizing a force field trained with a machine learning (ML) methodology, as ML has already proven itself as an effective tool for assisting quantum chemical research for chemical space exploration.^30,31^ After that, we discuss in more detail the stability of three isovalent substitutions, K^+^ and Ag^+^ for Na^+^ and Ga^3+^ for Al^3+^, as well as the competing substitutions into the corresponding ternary chlorides KAlCl_4_, AgAlCl_4_, and NaGaCl_4_. Finally, we explore the effect of a specific and promising aliovalent substitution, namely, using Zn^2+^ as a substitute for Al^3+^ in NaAlCl_4_, and the corresponding substitution of Al^3+^ in Na_2_ZnCl_4_.

## Computational Details

All calculations were performed with the plane-wave program package VASP^32−34^ and PAW pseudopotentials.^35^ A cutoff energy of 520 eV was used for PBE^36^ in accordance with the parameters employed by the Materials Project.^37^ The coordination of all calculations was done with the pymatgen Python package.^38^ The DFT-D3 dispersion correction^39^ with the Becke–Johnson damping^40^ was used in all calculations. The -point density was determined via a convergence of the KSPACING parameter in VASP to within 5 meV and adjusted accordingly for all supercells. In the production runs, structures were considered converged if the highest normed atomic force was lower than 0.003 eV/Å and the energy convergence of the SCF was lower than 1 × 10^–7^ eV to guarantee reasonably tight convergence for the phonon calculations with Phonopy.^41,42^ Additionally, the number of grid points for the FFT grid was raised to 12 Å^–1^*l*, where *l* corresponds to one of the lattice constants *a*, *b*, or *c*, to improve the accuracy of the phonon calculations and avoid artificial imaginary modes. The optimization of doped structures was done with the default energy convergence of VASP of 1 × 10^–4^ eV, force convergence at 0.01 eV/Å, and the default -point grid density determined by pymatgen.

The supercells for the phonon calculations were generated in a way such that the lattice parameters are greater than 12 Å, and symmetry was fully exploited in VASP and Phonopy.

All required structures were sourced as CIF files from the online database ICSD,^43^ and all optimized structures are provided in the Supporting Information in VASP POSCAR format. The program Supercell^44^ was used to generate the possible configurations for all investigated substitutions, and if necessary, the number of permutations obtained from each supercell calculation was limited to the 100 configurations with the lowest classical Coulomb energy. The structures of the most stable configurations are provided in the Supporting Information in VASP POSCAR format. In the case that extra sodium ions were required to balance the charge of the cell, the most stable positions for the additional ions were evaluated based on the Coulomb interactions with the rest of the structure. In the opposite case, sodium vacancies were introduced as partial occupations in the supercell input. In all cases where a doubled supercell was generated, the cell direction with the smallest cell vector was doubled. All structure images were generated with VESTA (version 3.5.8)^45^ and all diagrams were created using Matplotlib.^46^

### Machine-Learned Force Field

For machine learning-assisted phonon calculations, training for the machine-learned force fields (MLFF) was carried out on each system in two steps using default parameters for the force field fitting, with one exception. The weighting of the forces during training, specified by the parameter ML_WTIFOR, was increased from a default value of 1 to 2, since it was found to significantly reduce the training errors and ensures that the maximum root mean-square error of the forces remains below 15 meV/Å. It is half of the desired range of force errors for training as stated in the best practices for VASP^47^ and is well below the errors of other machine learning-based investigations,^48,49^ so it was deemed an acceptable error for the training of a property that heavily relies on accurate forces.

In the first step of the training, a molecular dynamic (MD) simulation was carried out at 400 K with a Langevin thermostat, friction coefficients of 1 ps^–1^ and 10 ps^–1^ for the ions and cell, and a time step of 2 fs. The temperature was chosen because it lies slightly below the melting point of the structures of interest, NaAlCl_4_ (MP at 426 K^22^), and is expected to yield more movement for better configuration sampling without disrupting the lattice through an actual change of state. The simulation was carried out over differing amounts of simulation steps to evaluate the influence of the training duration on the performance. VASP uses a Bayesian-learning algorithm to train a force field on the different configurations obtained during the MD simulation and estimates the expected errors for energies, forces, and stresses at every step. If the errors are within the set limits, then the simulation is propagated using the force field. However, if the errors exceed the set limits, the subsequent step is evaluated using an ab initio single-point calculation, and the force field is updated accordingly. The settings for these ab initio calculations are identical to the settings for the initial optimization of the structure in question. This approach allows for a fast sampling of configurations, as only configurations that deviate by a significant margin from already saved configurations are evaluated using costly ab initio calculations.

Once the MD simulation is complete, the information from all sampled configurations was refitted using VASP to accelerate the prediction of the forces.

## Results and Discussion

### Initial Substitution Tests

**Figure 1:**
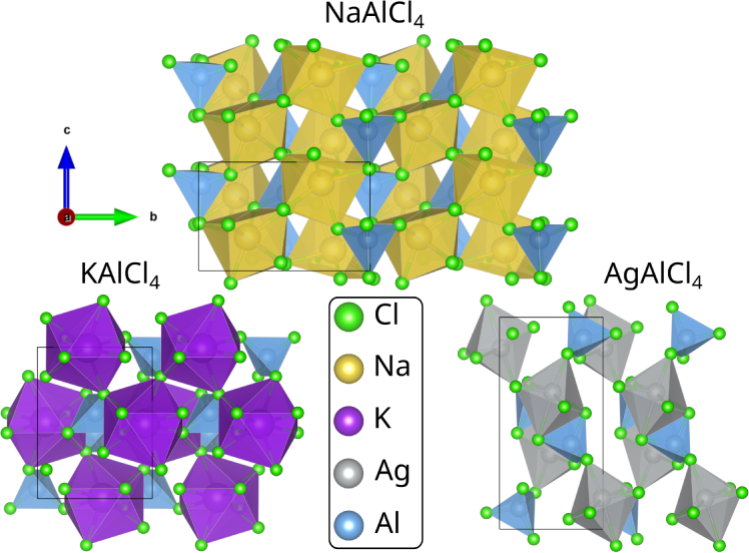
Geometry of NaAlCl_4_, KAlCl_4_, and AgAlCl_4_. The unit cell is indicated by a black box, and the view axis is along [100].

In the first step, the approximate stability of a few potential doping candidates for sodium and aluminum in NaAlCl_4_ was computed based on a substitution into the structure of NaAlCl_4_ (space group 19, *P*2_1_2_1_2_1_), which is shown in Figure 1.

The investigated doping candidates on the Na^+^ site consist of K^+^, Ag^+^, Mg^2+^, Ca^2+^, Sr^2+^, Ba^2+^, Sc^3+^, La^3+^, and Y^3+^, since these ions prefer an octahedral coordination in their respective chlorides.^50−57^ On the Al^3+^ site, Zn^2+^ and Ga^3+^ were considered because of their preference for tetrahedral coordination in their chlorides.^58,59^ In all cases, either one sodium ion or one aluminum ion was replaced with one of the respective substituents, and any discrepancy in charge was balanced by removing or adding sodium ions to the lattice. For the substitution with Y^3+^, La^3+^, and Sc^3+^, a 2 × 1 × 1 supercell was used because of the high amount of vacancies introduced into the lattice, yielding an effective substitution of 12.5% of all available positions for those ions. For all other ions, a unit cell was used for substitution, substituting 25% of all available positions. We list in Table 1 the resulting formation energies. We report the formation energies Δ*E*_bin_ directly from the binary chlorides ACl_*x*_/BCl_*x*_, NaCl, and AlCl_3_. Moreover, we also give the formation energy  per substitution, i.e., the formation energy for a substitution of Na^+^ with respect to the reaction precursors ACl_*x*_, AlCl_3_, and NaAlCl_4_, and for the substitution of Al^3+^ using BCl_*x*_, NaCl, and NaAlCl_4_, where A and B are one of the aforementioned substituents. This corresponds de facto to a doping of presynthesized NaAlCl_4_. For an exemplary substitution of Al^3+^ with Zn^2+^, the corresponding reaction would be 3NaAlCl_4_ + 2NaCl + ZnCl_2_ → Na_5_ZnAl_3_Cl_16_.

**Table 1:** Formation Energy of Substituted Structures per Substitution Compared to  (NaAlCl_4_ and the Binary Chlorides as Precursors) and to Δ*E*_bin_ (Only Binary Chlorides as Precursors)

dopant	substituted ion	/eV	/eV
K^+^	25% Na^+^	–0.42	–0.88
Ag^+^	25% Na^+^	0.09	–0.37
Mg^2+^	25% Na^+^	0.93	0.63
Ca^2+^	25% Na^+^	0.25	–0.05
Sr^2+^	25% Na^+^	0.19	–0.11
Ba^2+^	25% Na^+^	0.13	–0.18
Sc^3+^	12.5% Na^+^	1.76	1.00
Y^3+^	12.5% Na^+^	1.11	0.35
La^3+^	12.5% Na^+^	0.99	0.23
Zn^2+^	25% Al^3+^	0.55	0.09
Ga^3+^	25% Al^3+^	–0.34	–0.79

The ions that are chemically most similar to the ions they substitute yield the greatest stability compared to NaAlCl_4_, as every K^+^ that replaces a Na^+^ ion in the lattice generates −0.42 eV and every Ga^3+^ that replaces a Al^3+^ ion generates −0.34 eV. A direct synthesis from binary chlorides would be equally favorable. This suggests a high likelihood that either substitution can be carried out experimentally. From the remaining substitutions, Ag^+^ is another likely candidate since it only exhibits a low destabilization of 0.09 eV/substitution compared to NaAlCl_4_ and a stabilization of −0.37 eV compared to the binary chlorides. This substitution is likely because Ag^+^ has the same ionic charge and a similar ionic size as the substituted sodium, thus maintaining the electrostatic environment of the doped material.

The earth alkaline metals have a higher electrostatic charge than sodium, but the substitution of Na^+^ with barium, strontium, and calcium is only slightly less stable than the substitution with silver in the range of 0.13–0.25 eV. For increasing ionic radii,^60^ these elements exhibit a trend toward higher stability, with barium being the most stable and magnesium being the least stable at 0.93 eV per substitution. The same trend is observed regarding their stability with respect to those of the binary chlorides. The remaining substitution candidates for sodium, scandium, yttrium, and lanthanum are significantly unstable at 1.76, 1.11, and 0.99 eV, respectively. In their case, the instability most likely originates from the introduction of two vacancies per substituted ion into the lattice, their high charge, and their small ionic radius, as the smaller Sc^3+^ ion is even less stable than the Y^3+^ and La^3+^ ions.

As for the substitution of Zn^2+^ for Al^3+^, it has been found to be unstable, with an energy cost for doping of 0.55 eV. The differing charges of the ions and their diverging Shannon ion radii of 0.54 Å for Al^3+^ and 0.74 Å for Zn^2+^ are likely the reason for the instability.^60^ Due to the low relative stability of ZnCl_2_, the formation from the binary chlorides is, however, almost favorable at 0.09 eV, and it appears to be the only potentially feasible aliovalent substitution.

In summary, the substitutions of sodium and aluminum with their respective elemental counterparts in the row below them in the periodic table lead to the most stable results. The stability seems loosely linked to the difference in charge and ion size, as substituents that deviate the most from the substituted ion are generally the least stable. The exceptions are the earth alkaline metals, where the stability rises with ion size.

### Phonon Calculations with Machine Learning

Vibrational thermodynamics are one potential source of stability for the substitution of NaAlCl_4_, but the phonon calculations required to obtain the contribution of the vibrational energy to the total energy are significantly more expensive than the initial geometry optimizations for most structures, especially larger ones with low symmetry, as the required amount of displacements rises with the number of atoms and degrees of freedom inside the investigated structure. Substituted structures suffer the most from this because they usually consist of low-symmetry supercells of the original structure. For example, the initial optimization of the most stable configuration of NaAl_0.5_Ga_0.5_Cl_4_ took 22 400 s for 90 optimization steps, whereas the phonon calculation required 722 000 s over 72 displacements on the same machines (2× AMD Epyc 7302 16c CPU, 32 cores total). To address this tremendous computational effort, we explored how well the on-the-fly machine-learned force fields (MLFF) implemented in VASP can reproduce the results of a full ab initio phonon calculation for a test set of structures comprising NaCl, KCl, AgCl, ZnCl_2_, GaCl_3_, and AlCl_3_, as well as their experimentally documented mixed chlorides and all theoretical mixed structures with a 50% substitution rate of either Na^+^ or Al^3+^.

The MLFF was trained on a molecular dynamics (MD) simulation of the unit cell at 400 K with 100, 250, 500, and 1000 simulation steps, taking into account the low melting point of NaAlCl_4_ of 426 K.^22^ The results and timings for the trained force fields are compared to ab initio phonon calculations for same-sized supercells in Table 2 at 300 K.

**Table 2:** Average and Maximum Relative Prediction Error (%) of the Vibrational Thermodynamic Corrections at 300 K  Obtained by MLFF-Assisted Phonon Calculations (Using the Force Field Generated after 100, 250, 500, and 1000 MD Training Steps) from the Result for Ab Initio Phonon Calculations . Corresponding Training Times are Provided in Seconds and Relative to the Ab-Initio Phonon Calculations at a Total Computation Time of 5.29M Seconds

	(*E*_vib_^MLFF^ – *E*_vib_^ab-initio^)/*E*_vib_^ab-initio^ at 300 K			
MD simulation steps	Average/%	Maximum/%	Abs. time, s	Rel. time %
100	2.3 ± 3.0	9.5	164 000	3.1
250	1.4 ± 1.5	4.5	266 000	5.0
500	0.9 ± 1.3	3.3	464 000	8.8
1000	0.8 ± 1.3	4.3	759 000	14.4

A more detailed list of the results and the training errors of the force field can be found in the Supporting Information in Figures S1–S6 and as data in xlsx format.

In summary, the maximum prediction error of the force field remains within 10% of the ab initio calculation even for short training with just 100 MD training steps, yielding an average error of 2.3 ± 3.0%.

**Figure 2:**
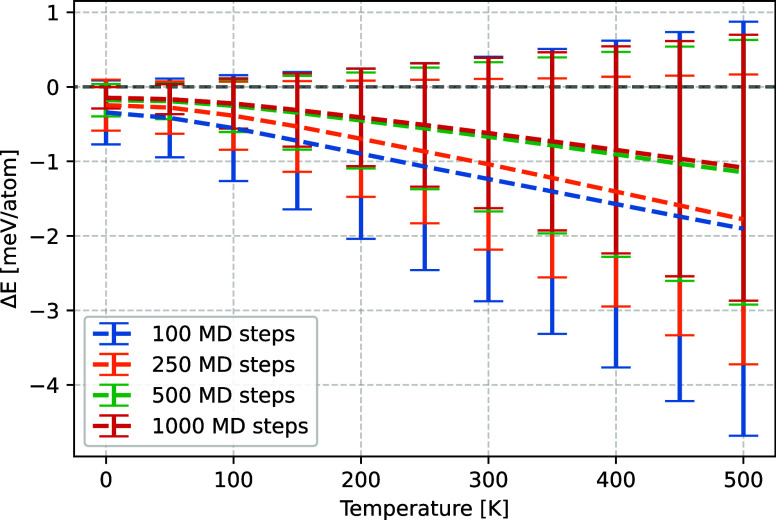
Absolute average deviation of the vibrational energies at different temperatures that were obtained from phonon calculations with on-the-fly machine-learned force fields to an ab initio phonon calculation. The different colors indicate different amounts of MD simulation steps used for training the corresponding force fields, as precised in the Computational Details.

In absolute terms, as shown in Figure 2, the prediction error at 300 K amounts to −0.7 ± 1.0 meV/atom for 500 MD training steps, which remains within the chemical accuracy of ∼43 meV^61^ for cells up to 30 atoms and well within the regular errors of GGA-level DFT. Parity plots for the prediction errors of all investigated structures at 300 and 500 K are shown in Figures S7 and S8. Both the absolute and relative prediction errors improve from 100 to 500 training steps, but no significant accuracy is gained by using 1000 training steps. All training times undercut the computation time for the ab initio phonon calculations by an order of magnitude.

The main time saving is gained for the doped systems due to their large size and low symmetry. For example, the calculation of all displacements with PBE-D3(BJ)/500 eV for NaAl_0.5_Ga_0.5_Cl_4_ took 722 000 s, whereas the whole process of fitting the force field and calculating all displacements with it took 29 000 s for 500 MD steps, which accounts for a speed-up of about 25 times. In these cases, the machine learning process likely benefits from the simultaneous displacement of all atoms of the structure in the MD simulation, which allows efficient sampling of the interatomic forces.

Even small systems with high symmetry benefit from the usage of the MLFF. For example, only two displacements need to be calculated for NaCl, which takes 12 400 s with PBE, whereas the fitting of the MLFF takes 3200 s, still gaining a speed-up of a factor of 3. Here, the machine learning process is likely accelerated by the low complexity of the system. The force-field training takes up the largest share of the computation time, about 92% at 100 steps and 98% at 1000 steps, whereas the actual calculation of the displacements with the force field takes <1% in all cases.

In summary, training a force field with the machine learning algorithm implemented in VASP significantly accelerates the calculation of vibrational thermodynamics compared to a pure ab initio calculation at deviations of about 0.9 ± 1.3% and −0.7 ± 1.0 meV/atom at 300 K and 500 training steps, respectively. The amount of training steps necessary for an adequate result does not depend on the size and symmetry of the investigated system, and training on an MD simulation with 500 training steps appears to be a good compromise between accuracy and cost.

### Configurational Entropy

Configurational entropy is another significant source of stabilization for mixed compounds such as the substituted structures investigated in this work. The value of the configurational entropy *S*_conf_ was calculated with the formula
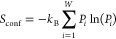
1where *W* is the amount of possible configurations and *P*_*i*_ is the probability of each configuration *i*. Assuming the same energy for all states, *P*_*i*_ becomes 1/*W*, and the formula can be rearranged to the formula for perfect mixing entropy of two components,
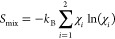
2where the fraction χ_1_ represents the relative amount of ion type 1 and χ_2_ is the relative amount of ion type 2 occupying the same site. Employing their relationship χ_1_ = 1 – χ_2_, the final formula for the entropy of a two-component mixture becomes
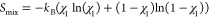
3Equation 3 effectively describes the maximum configurational entropy accessible for a two-component mixture.

### Isovalent Substitutions

In this part, the three most promising substitutions in NaAlCl_4_, i.e., of Na^+^ with K^+^ or Ag^+^, and of Al^3+^ with Ga^3+^ are discussed in greater detail, including an investigation of their stability compared to their precursors and the inclusion of entropic contributions, namely, vibrational and configurational effects.

The substitution of gallium into NaAlCl_4_ is straightforward, as both NaAlCl_4_ and NaGaCl_4_ share the same crystal structure (s.g. 19, *P*2_1_2_1_2_1_, see Figure 1) with nearly the same unit cell parameters (see Table S1).^62^ The calculated lattice parameters underestimate the measured parameters by about 1.3 ± 0.7% with the discrepancy most likely originating from the thermal expansion of the crystalline solids at room temperature. Moreover, the bond between Al^3+^/Ga^3+^ and Cl^–^ is significantly shorter than that expected from their Shannon ionic radii. Instead of 2.35 Å for Al–Cl and of 2.43 Å for Ga–Cl, they are 2.16 and 2.20 Å, respectively, indicating a significant covalent contribution to the bond.

**Figure 3:**
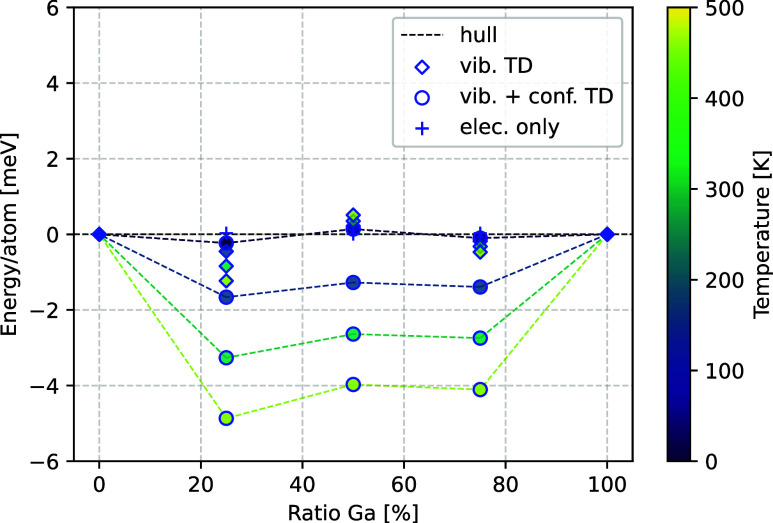
Formation energy diagrams of NaAlCl_4_ and NaGaCl_4_. Crosses indicate the stabilization obtained from purely electronic energies, while diamonds take vibrational thermodynamics into consideration (vib. corrected), and circles moreover consider the configurational entropy (vib. + conf. TD) assuming a solid solution using eq 3. For the sake of clarity, temperatures higher than 450 K are omitted due to the low melting point of NaAlCl_4_ of 426 K.^22^

The ionic substitution was performed for three concentrations, 25%, 50%, and 75%, and only based on the unit cell, yielding the formation energy diagrams shown in Figure 3.

Not considering any thermodynamic contributions, none of the doped structures is more stable than the mixture of NaAlCl_4_ and NaGaCl_4_, but the instability is in the order of <0.1 meV/atom. The stability range increases to values in the range of −1.5 to 0.5 meV/atom if vibrational thermodynamics is considered, yielding a shallow hull that is below the typical errors of PBE^63^ and the machine-learned force fields.

The configurational entropy was estimated based on the calculations done for the unit cell and the 2 × 1 × 1 supercell of the 1:1 mixture. An identical stability was obtained for all investigated configurations, suggesting random occupation of the Al sites with aluminum and gallium ions. This is further corroborated by the local geometry, as the effective size of Al^3+^ and Ga^3+^ deviates by less than 2%, and the fact that both chlorides assume the same structure.

Assuming that eq 3 takes full effect for the mixture, the hull between NaAlCl_4_ and NaGaCl_4_ is dominated by it, resulting in a maximum stabilization of −5 meV/atom at 25%. Regarding the lattice parameters (Table S1), the doped structures do not deviate significantly from a linear interpolation from NaAlCl_4_ to NaGaCl_4_ and the Al–Cl and Ga–Cl bonds exhibit the same length as in the precursor structures. In conclusion, the intermediate structures NaAl_1–_*_x_*Ga_*x*_Cl_4_ were found to be more stable than the basic ternary end-member chlorides, with most of the stabilization originating from configurational entropy. Aluminum and gallium ions most likely undergo perfect mixing (solid solution) on the same lattice site.

**Figure 4:**
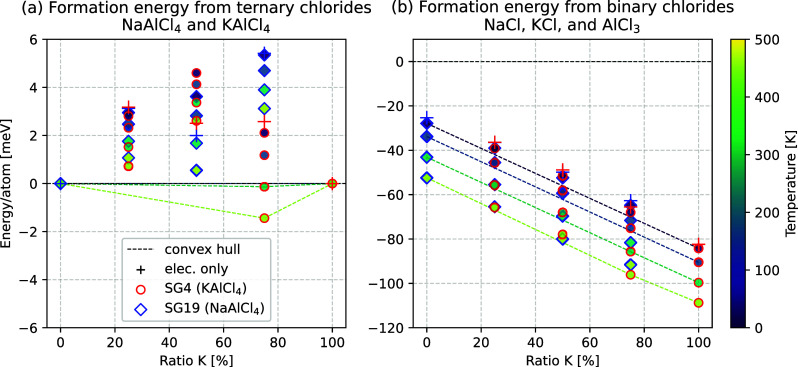
(a) Formation energy of K_*x*_AlCl_4_ from the ternary chlorides NaAlCl_4_ and KAlCl_4_. (b) Formation energy of K_*x*_AlCl_4_ from the binary chlorides NaCl, KCl, and AlCl_3_. Crosses indicate the stabilization obtained from purely electronic energies, while diamonds represent the stabilization under consideration of vibrational thermodynamics. Structures based on NaAlCl_4_ have a blue outline, and structures based on KAlCl_4_ have a red one.

The substitution of potassium for sodium in NaAlCl_4_ is also isovalent, yielding the general sum formula Na_1–_*_x_*K_*x*_AlCl_4_, of which the ratios *x* = 0.25, 0.5, and 0.75 were investigated. However, the structure of KAlCl_4_ is at variance to NaAlCl_4_, as it belongs to the space group 4 (*P*2_1_, see Figure 1).^64^ Therefore, both the substitution of K into NaAlCl_4_ and the corresponding Na substitution into the KAlCl_4_ structure need to be considered. The computationally obtained phase diagram for the Na_1–_*_x_*K_*x*_AlCl_4_ system is shown in Figure 4.

Substituting the structure of KAlCl_4_ with sodium and the structure of NaAlCl_4_ with potassium yields structures with a formation energy from the ternary chlorides in the range between 2 and 6 meV/atom at low temperatures (Figure 4a). Due to vibrational thermodynamics, the mixtures become significantly more stable at higher temperatures, with a 25:75 ratio of Na:K reaching a stability of −1.5 meV/atom. As with the substitution with gallium, the absolute stability is within the error of the methodology, but the stabilizing effect of the vibrational thermodynamics is about twice the error of the machine-learned phonon calculations. Configurational entropy is expected to lower the energies shown in Figure 4 further, but the evaluation is complicated by the existence of two competing structures and a nonzero energy discrepancy between the different configurations. For the unit cell configurations, this discrepancy is in the order of 0.05 eV for structures based on KAlCl_4_ and of 0.09 eV for structures based on NaAlCl_4_, recovering 94% and 81% of the maximum possible entropy at 300 K, respectively. As such, the configurational entropy is expected to become large enough to stabilize the 75% substituted structure beyond the errors of the methodology and potentially stabilize other ratios as well. At the same time, the mixtures and ternary chlorides are energetically highly favorable compared to the binary chlorides, as their electronic formation energy from those ranges from −25 for NaAlCl_4_ to −65 meV/atom for KAlCl_4_ (Figure 4b), which is an order of magnitude higher than the instability of the mixtures from the ternary chlorides. Their energies are further lowered by favorable contributions from their vibrational thermodynamics. With the formation energy of KAlCl_4_ from its binary chlorides being about 40 meV/atom lower than the formation energy of NaAlCl_4_, mixtures with a higher amount of potassium are expected to be favored in synthesis.

In summary, compositions K_*x*_AlCl_4_ are slightly unstable compared to NaAlCl_4_ and KAlCl_4_ as precursor structures, but highly stable compared to NaCl, KCl, and AlCl_3_ as precursors, indicating a very likely metastable existence as a mixed phase.

**Figure 5:**
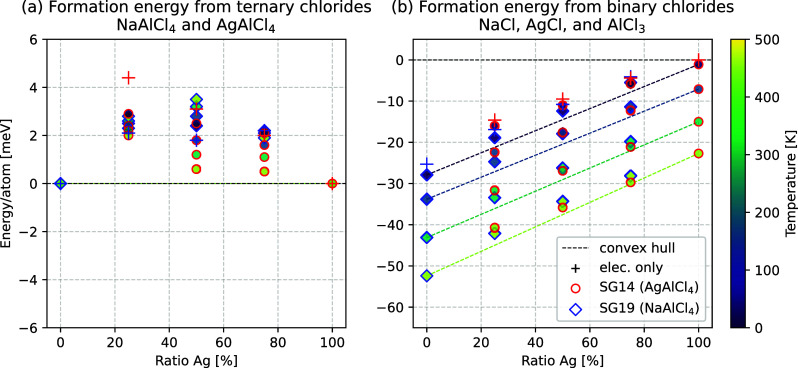
(a) Formation energy of Ag_*x*_AlCl_4_ from the ternary chlorides NaAlCl_4_ and AgAlCl_4_. (b) Formation energy of Ag_*x*_AlCl_4_ from the binary chlorides NaCl, AgCl, and AlCl_3_. Crosses indicate the stabilization obtained from purely electronic energies, while diamonds represent the stabilization under the consideration of vibrational thermodynamics. Structures based on NaAlCl_4_ have a blue outline, and structures based on AgAlCl_4_ have a red one.

Another meaningful isovalent substituent for sodium is silver, according to Ag_*x*_AlCl_4_, and the ratios and substitutions are identical to the investigation for potassium as a dopant, with the difference that AgAlCl_4_ has the space group 14 (*P*2_1_/*c*, see Figure 1).^65^ The resulting formation energy diagrams are shown in Figure 5.

Substituting Ag^+^ into NaAlCl_4_ or Na^+^ into AgAlCl_4_ yields slightly more stable results than the substitution with potassium, with all structures having a formation energy from the ternary chlorides in the range of 0.5–4 meV/atom (Figure 5a). The difference is likely explained by the fact that Ag^+^ is more similar to Na^+^ than K^+^. For once, the Shannon radius of Ag^+^ is only 13% greater than that of Na^+^, whereas K^+^ is about 35% larger, making the former a better fit for each other.^60^ The investigated unit cell configurations support a greater exchangeability of Na^+^ with Ag^+^ in contrast to K^+^ due to the better match of their ionic radii as well, as the configurations based on AgAlCl_4_ deviate by just 0.01 eV and those based on NaAlCl_4_ by 0.02 eV. Consequently, the configurational entropy is expected to further stabilize the mixed structures Ag_*x*_AlCl_4_ by up to 5 meV/atom, as obtained for *x* = 0.5, approximating a solid solution with eq 3. The formation of the ternary chlorides and their mixtures from the binary chlorides, however, is the opposite of what is observed for potassium as a substituent, ranging from −25 meV/atom for NaAlCl_4_ to 0 meV/atom for AgAlCl_4_ (Figure 5b). Vibrational thermodynamics do stabilize all structures by another 15 meV/atom at room temperature, while the relative stability of NaAlCl_4_ is expected to yield a more favorable substitution of sodium into AgAlCl_4_.

In conclusion, the substitution Ag_*x*_AlCl_4_ yields a metastable phase based on the structure of AgAlCl_4_, yet it is expected to be synthesizeable from the binary chlorides, with a preference toward compounds with higher sodium content.

### Aliovalent Substitutions with Zinc

**Figure 6:**
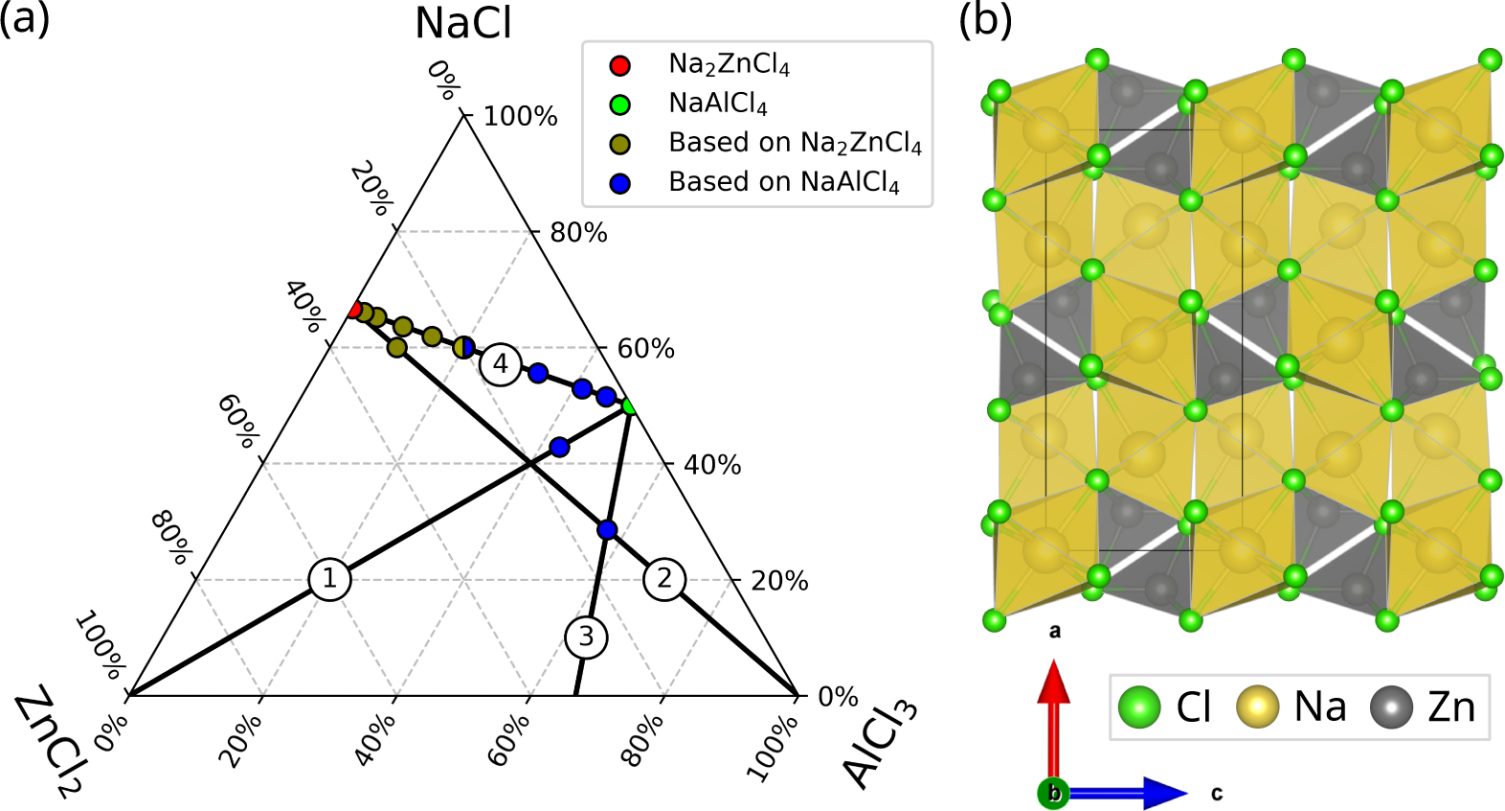
(a) Phase diagram of all calculated mixtures of NaCl, ZnCl_2_, and AlCl_3_. The labeled axes indicate the (hypothetical) end points of the four investigated types of substitution. (b) Structure of Na_2_ZnCl_4_ viewed along the [010] axis. The unit cell is indicated by a black rectangle.

To understand how substitutions with ions of different charge perform, both cationic sites in NaAlCl_4_ were substituted with varying amounts of zinc ions and, vice versa, the zinc sites in Na_2_ZnCl_4_ were substituted with aluminum ions. In both cases, the amount of Na was also adjusted to properly compensate for the charge. Na_2_ZnCl_4_ has the space group 62 (*Pnma*, see Figure 6b), which means that it is essentially a olivine-type structure analogous to the well-known cathode material LiFePO_4_, where Na takes the positions of both Fe and Li, and ZnCl_4_ groups replace PO_4_ ones. Zinc was predominantly chosen because it prefers a tetrahedral coordination like aluminum as chloride^58,66^ and because the substitution of 25% of the aluminum ions with zinc ions was found to have little instability compared to the binary chlorides, in this case by 0.09 eV per substitution. The investigated mixtures are shown in the phase diagram (PD) in Figure 6a, and all energies are given in reference to a mixture of NaAlCl_4_ and Na_2_ZnCl_4_ plus any of the binary chlorides if necessary.

The least stable result is obtained for structures based on a hypothetical binary reaction of ZnCl_2_ + 3NaAlCl_4_ → Na_3_ZnAl_3_Cl_14_ (line 1 connecting NaAlCl_4_ and ZnCl_2_ in Figure 6a), assuming that one-fourth of all aluminum ions were substituted by zinc ions in the lattice of NaAlCl_4_. Accordingly, two sodium ions and two chlorine ions had to be subtracted from the unit cell, yielding a destabilization of 1.36 eV per substituted aluminum. Similarly, the binary reaction AlCl_3_ + 3Na_2_ZnCl_4_ → Na_6_Zn_3_AlCl_15_ (line 2 connecting Na_2_ZnCl_4_ and AlCl_3_ in Figure 6a), assuming that one-fourth of all zinc ions in the lattice of Na_2_ZnCl_4_ are substituted with aluminum ions, yields a destabilization of 0.85 eV per substituted aluminum. In this case, only one sodium ion and one chlorine ion were subtracted from the unit cell. The main source of instability in these simple models is the retention of the structures of NaAlCl_4_ and Na_2_ZnCl_4_, which necessitates the removal of several ions in the lattice, especially of two and one chlorine anions per unit cell, respectively. More stable structures with different ratios and space groups may exist but were not the subject of this study and require a more involved global minimum search.

One possible option for a substitution that affects only the number of cations is the replacement of sodium ions with zinc ions, which was investigated for NaAlCl_4_. Due to the different charge of Na^+^ and Zn^2+^, this introduces one vacancy for every two substituted sodium ions, according to . The reaction formula for this hypothetical reaction is ZnCl_2_ + 2AlCl_3_ + 2NaAlCl_4_ → Na_2_Zn(AlCl_4_)_4_ and the corresponding line 3 in the PD aims toward the composition ZnAl_2_Cl_8_, which to the best of the authors’ knowledge has not been experimentally observed. This substitution yields a destabilization of 0.97 eV per unit cell, indicating that the introduction of vacancies and zinc ions into the sodium sites is unfavorable.

Adding NaCl to the previously discussed binary reactions to compensate for the loss of chlorine ions in the lattice yields the structures located on line 4 between Na_2_ZnCl_4_ and NaAlCl_4_ in the PD in Figure 6a. If existing as single phases, these could be written as . Unlike the previous combinations, these yield significantly more stable structures and were consequently investigated for a larger range of ratios and cell sizes to evaluate the influence of those parameters on the calculated stability.

**Figure 7:**
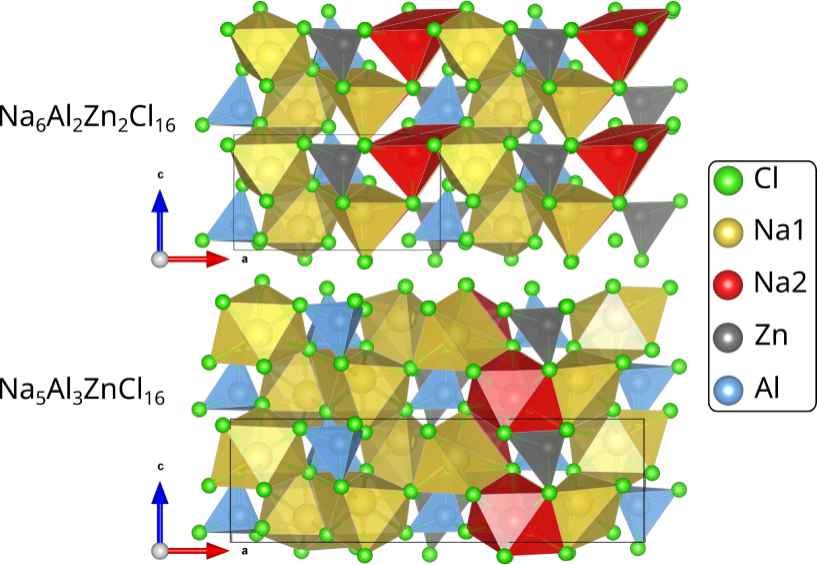
Most stable substituted NaAlCl_4_ structures at 25 and 50% Zn^2+^ substitution. Both substituted structures are viewed along the same axis [010]. Yellow polyhedra represent sodium on regular lattice sites, and red polyhedra represent sodium on interstitial sites.

**Figure 8:**
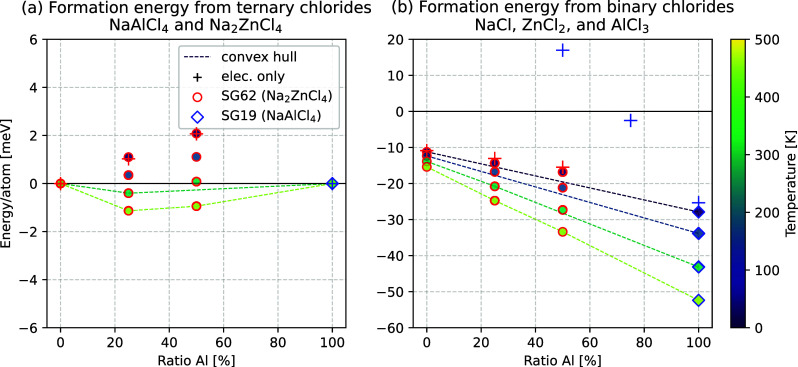
(a) Formation energy from the ternary chlorides for mixtures of Na_2_ZnCl_4_ and NaAlCl_4_. (b) Formation energy from the binary chlorides for the mixtures of Na_2_ZnCl_4_ and NaAlCl_4_. The results are for the structures shown in Figures 7 and 9 with vibrational thermodynamics and configurational entropy (circles) and without (crosses) for substituted structures based on Na_2_ZnCl_4_ (red) and NaAlCl_4_ (blue). Due to their general electronic instability (blue crosses), no thermodynamic properties were calculated for the mixtures based on NaAlCl_4_, and the formation energies are only shown for the formation from binary chlorides. A rescaled diagram for the formation energy from the ternary chlorides can be found in Figure S9.

For the substitution of Al^3+^ with Zn^2+^ in NaAlCl_4_, the most stable state is found for a ratio of Zn:Al of 50:50 according to Na_2_ZnCl_2_ + NaAlCl_4_ → Na_3_ZnAlCl_8_ with an energy of 0.89 eV (34 meV/atom) per unit cell and 0.45 eV per substituted aluminum ion compared to a mixture of NaAlCl_4_ and Na_2_ZnCl_4_, and it is shown in Figure 7. The stability compared with that of the binary chlorides is about 0.44 eV (17 meV/atom), as shown in Figure 8b.

The most likely reason for the remaining destabilization is the addition of Na^+^ ions to the interstitial sites to balance the charges. The most stable position for the extra ion was found to correspond with the experimental findings for pure NaAlCl_4_^25^ and is indicated with red spheres and coordination polyhedra in Figure 7. The stability does improve at lower substitution ratios, as the most stable structure with a 25:75 ratio is only 0.47 eV (18 meV/atom) less stable per unit cell and per substituted aluminum ion than a corresponding mixture of NaAlCl_4_ and Na_2_ZnCl_4_ and 0.06 eV (2 meV/atom) more stable per unit cell than a corresponding mixture of binary chlorides, as shown in Figure 8b. Due to the differing size and charge of Zn^2+^ and Al^3+^ ions, they arrange themselves in a clear order instead of allowing for a random distribution across the original aluminum sites, which is apparent in both ratios shown in Figure 7. The ratio 25:75 (Figure 7) shows the ordering most clearly, as it features unique layers perpendicular to the axis containing all zinc and excess sodium ions, which are separated by the regular NaAlCl_4_ bulk structure.

The size of the substituted unit cell exceeds the size of the pristine cell due to the additional sodium ions and the larger ionic radius of Zn^2+^ compared to Al^3+^. This shows itself mostly as an expansion along the *a* axis (see Table S2). Ultimately, a substitution of aluminum in NaAlCl_4_ with zinc is unlikely since it is significantly less stable than the ternary precursors but indicates that the different charges benefit ordering.

**Figure 9:**
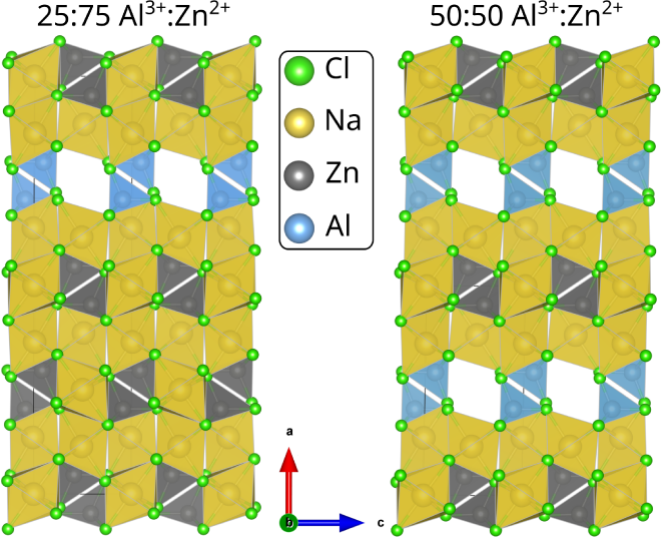
Most stable substituted Na_2_ZnCl_4_ structures at 25% and 50% Al^3+^ substitution. Both substituted structures are viewed along the same axis [010].

The same fundamental behavior is observed for the substitution of Al^3+^ into Na_2_ZnCl_4_ along line 4 in the phase diagram in Figure 6. The most stable substituted structures are similarly composed of different layers, but they are energetically much more favorable than the previously discussed substitution of NaAlCl_4_, likely due to the fact that the substitution introduces vacancies to existing lattice sites rather than adding Na^+^ ions to secondary, less stable lattice sites. Accordingly, the most stable structure for a 50:50 ratio of Zn^2+^ to Al^3+^ has a stability of 0.17 eV, while a stability of 0.10 eV is observed for a 75:25 ratio in the preliminary optimization. The stable structures are listed in Figure 9. They all contain aluminum ions and sodium vacancies in a separate layer along the *a*-axis of the crystal structure.

Due to their low instability, a full relaxation and machine learning-assisted phonon calculation were carried out for these structures. Upon complete relaxation, the stabilization energy with respect to the ternary chlorides decreased further to 0.05 (2 meV/atom) and 0.03 eV (1 meV/atom) for 50% and 25% substitutions of Al in Na_2_ZnCl_4_, respectively, as shown in Figure 8a.

The inclusion of vibrational thermodynamics and configurational entropy further stabilizes the structures at higher temperatures, yielding a Gibbs free reaction energy of 0 eV between 300 and 400 K and above that a shallow convex hull for the 75:25 mixture. The 50:50 mixture is only slightly less stable. In both cases, the only relevant contribution to the configurational entropy (>99% as calculated with eq 1) originates from the multiplicity of the most stable state, which amounts to about −1 meV/atom at 450 K in both cases. The influence of vibrational thermodynamics is about −2 meV/atom at 450 K, which is twice that of the configurational entropy.

The most likely reason for the relatively low instability of these substituted structures originates from the ion distribution, as each layer of the structure has the same number of positive charges. In addition, it allows the surroundings of the aluminum ion to relax according to its smaller size, yielding a layer that is 0.3 Å smaller than the average interlayer distance of 3.45 Å. This also shows itself as a contraction along the *a* axis (see Table S2), contrasting the expansion of NaAlCl_4_ due to its substitution with zinc. Similar to the previously discussed formation energy diagram for the mixtures of NaAlCl_4_ with AgAlCl_4_ (Figure 5), the formation energy from the binary chlorides NaCl, ZnCl_2_, and AlCl_3_ is strongly negative, especially toward a higher content of Al^3+^ (Figure 8b). This indicates that a metastable, aluminum-substituted intermediate based on the structure of Na_2_ZnCl_4_ may be possible. In any case, a composition close to those of Na_1.75_Zn_0.75_Al_0.25_Cl_4_ and Na_1.5_Zn_0.5_Al_0.5_Cl_4_ appears to be synthesizeable.

## Conclusions

In summary, a range of substitution options for NaAlCl_4_ was found with density functional theory on the GGA level. Of all candidates, potassium and silver are the most promising substitutes for sodium in NaAlCl_4_, yielding a low instability in the range of <4 meV/atom. Gallium is the most promising substitute for aluminum and possibly results in a full solid solution, due to their chemical similarities and same charge, and benefits from configurational entropy. The main factors deciding the stability of the substituted structures are the ion size, charge difference between the substituted ion and the substituent, and matching-preferred coordination spheres.

For a subset of all investigated structures, the vibrational thermodynamics was additionally assessed with the machine-learned force field as implemented in VASP, yielding a computational speed-up of about 11 times at an accuracy of about 0.9 ± 1.3% or −0.7 ± 1.0 meV/atom at 300 K for 500 training steps. The contribution of vibrational thermodynamics was found to be small but nevertheles significant , and the application of machine learning significantly improves the speed of the calculation of such contributions with a minor loss in accuracy. The performance for more demanding structures, such as cathode materials containing open-shell transition metals, is a valuable subject for future research as the fast and accurate calculation of thermodynamic contributions facilitates the search for stable and metastable phases.

Finally, the investigation into the chemical space between NaCl, ZnCl_2_, and AlCl_3_ revealed a potentially stable structure type of composition  based on the substitution of aluminum into Na_2_ZnCl_4_ with a layered configuration of the dopants along the *a*-axis comprising layers of sodium, sodium/zinc, and aluminum/sodium vacancies. Even though we did not investigate this directly, it is likely that a solid solution of aluminum in the Na_2_ZnCl_4_ structure exists for low substitution ratios and that the introduction of sodium vacancies improves the ionic conductivity. Experimental verification of these materials and their conductivity is a focus of ongoing research in our group.

## Supporting Information Available

All optimized structures in the VASP POSCAR format (TXT)
The results of the MLFF-assisted phonon calculations with computation times and energies (XLSX)
The methodology for the calculations and modeling, details of the structural similarity of NaAlCl_4_ and NaGaCl_4_, and the shift in lattice parameters due to substitution for NaAlCl_4_ and Na_2_ZnCl_4_ (PDF)
