# Supplementary material for: Exploring Cationic Substitutions in the Solid Electrolyte NaAlCl4 with Density Functional Theory
Source: J Phys Chem C Nanomater Interfaces. 2024 Nov 15;128(47):19978–88. doi: 10.1021/acs.jpcc.4c05559 (PMC11613591; doi:10.1021/acs.jpcc.4c05559)
Supplement: Supplementary file 3 — jp4c05559_si_003.pdf [file jp4c05559_si_003.pdf]

Exploring Cationic Substitutions in the Solid Electrolyte $NaAlCl_4$ with Density Functional Theory

Supporting Information

Michael Häfner and Matteo Bianchini*

Faculty of Biology, Chemistry and Earth Sciences, Universität Bayreuth, BAT, Weiherstraße 26, 95448 Bayreuth, Germany

E-mail: matteo.bianchini@uni-bayreuth.de

Phone: +49 (0) 921 / 55-4900

Performance of the Machine Learned Force Fields

Training Errors

In the following Figures S1, S2, S3, S4, S5, and S6, the root mean-square errors for the energies, forces, and stresses are plotted for the training runs with the most MD simulation steps for each investigated structure.

Figure S1: **Training errors for the binary chlorides.**

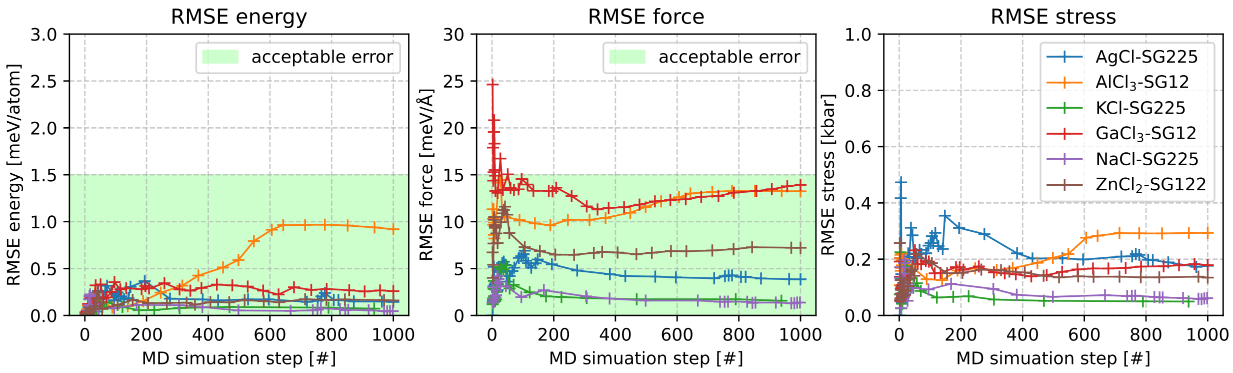

The crosses indicate the steps at which the force field was updated with ab-initio data.

Figure S2: **Training errors for the ternary chlorides.**

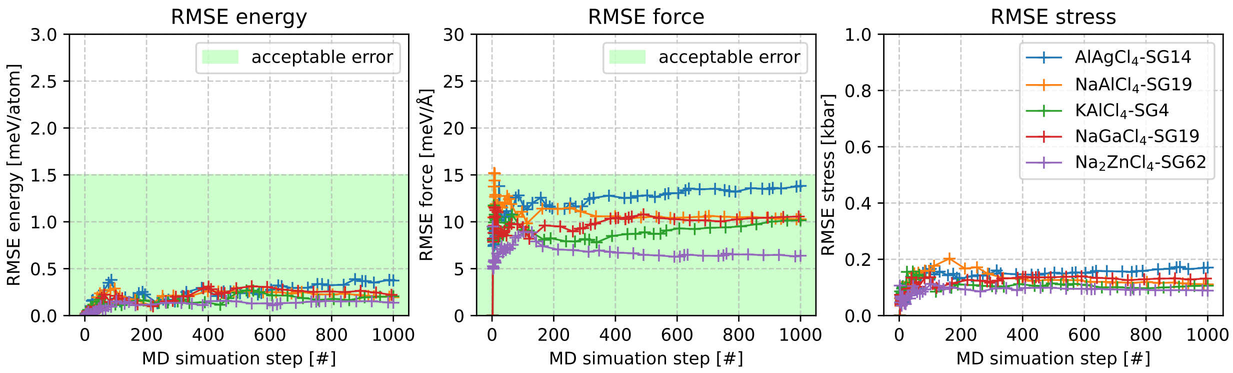

The crosses indicate the steps at which the force field was updated with ab-initio data.

Figure S3: **Training errors for mixtures of $NaAlCl_4$ and $KAlCl_4$.**

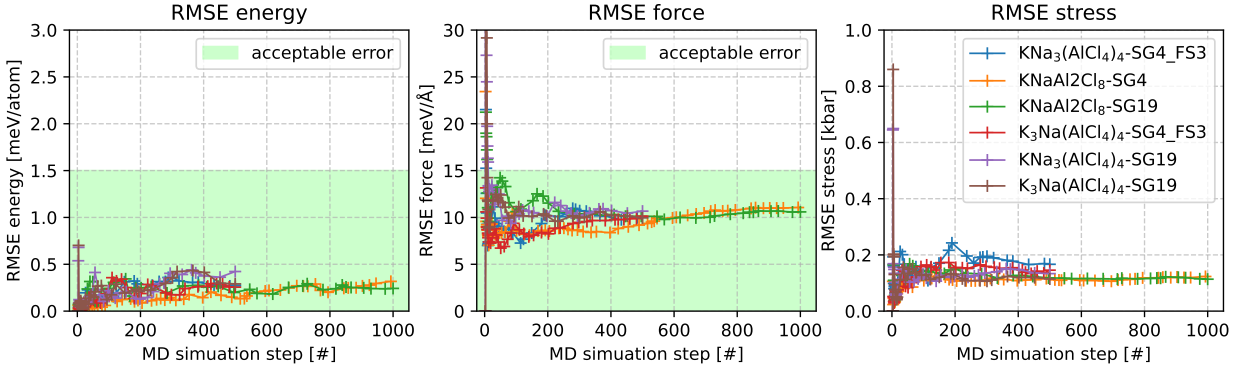

The crosses indicate the steps at which the force field was updated with ab-initio data.

Figure S4: **Training errors for mixtures of $NaAlCl_4$ and $AgAlCl_4$.**

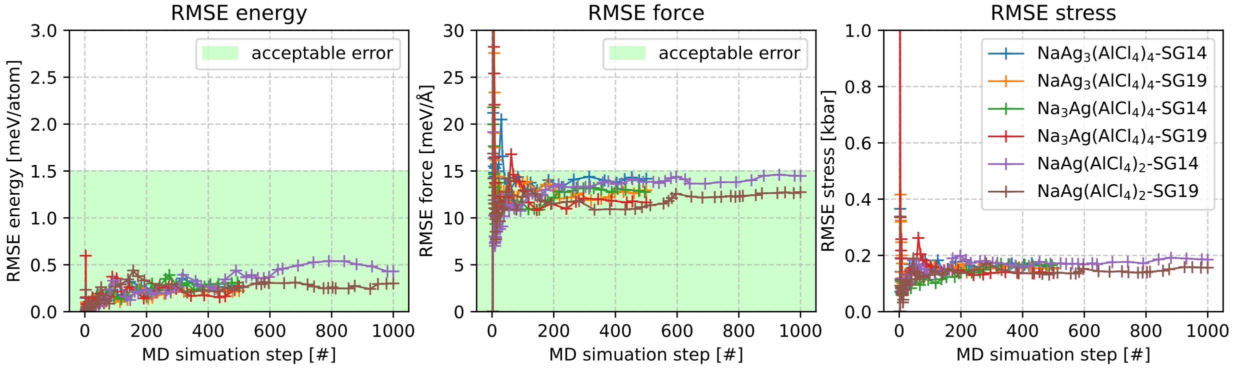

The crosses indicate the steps at which the force field was updated with ab-initio data.

Figure S5: **Training errors for mixtures of $NaAlCl_4$ and $NaGaCl_4$.**

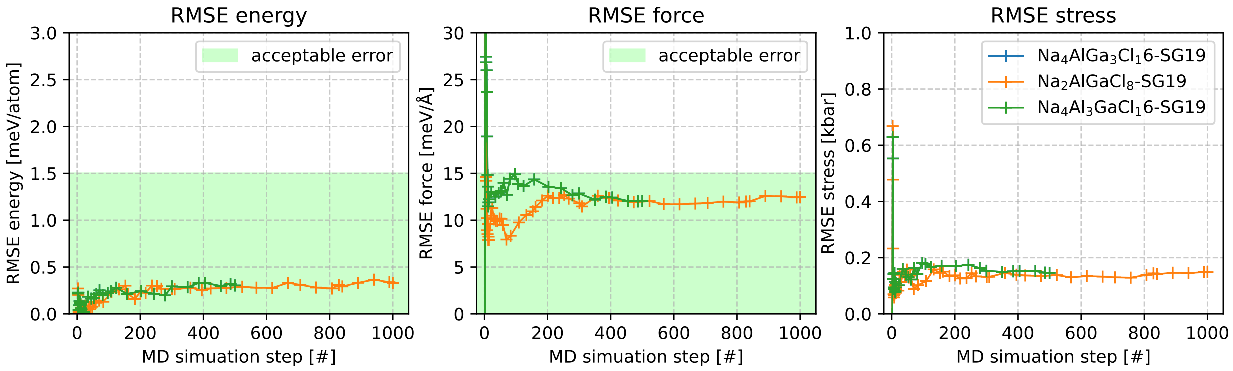

The crosses indicate the steps at which the force field was updated with ab-initio data.

Figure S6: **Training errors for mixtures of $NaAlCl_4$ and Na_2ZnCl_4.**

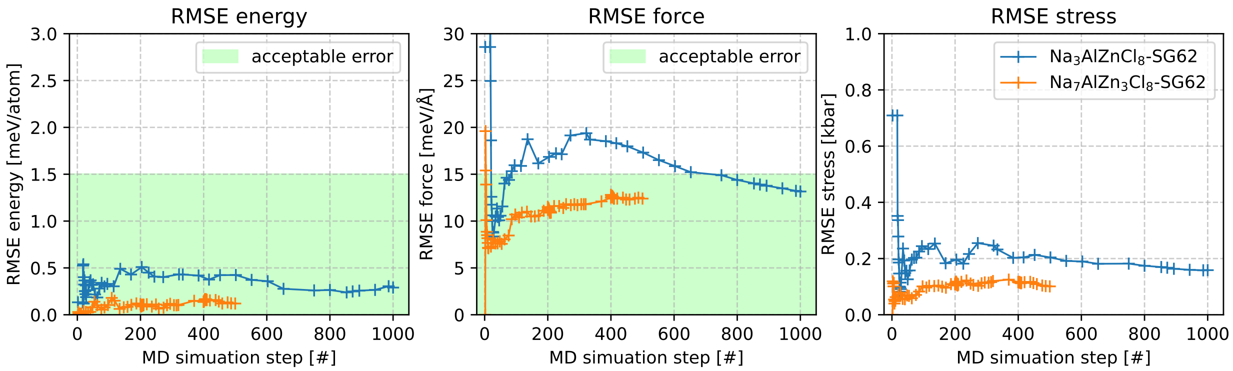

The crosses indicate the steps at which the force field was updated with ab-initio data.

All training simulations were run with the same set of parameters, except for the mixed structures $KNa_3(AlCl_4)_4$ and $K_3Na(AlCl_4)_4$ in the space group 4 of $KAlCl_4$ (Figure S3), which were run with `ML_WTIFOR = 3` due to large errors with a lower value for the force weighting.

Parity Plots for Phonon Calculations

In addition to the training errors, the errors for the vibrational energy corrections are plotted as parity plots (Figures S7 and S8) for the results at 300 K and 500 K.

Figure S7: **Parity plot for vibrational energy correction ΔE_{vib} at 300 K.**

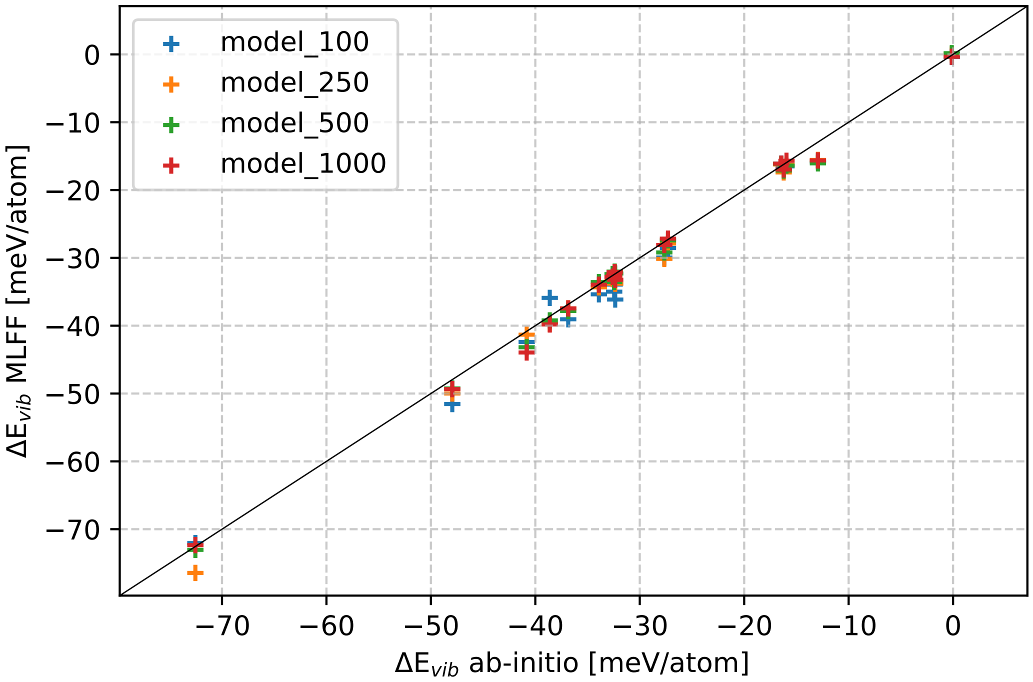

Parity plot of results for the machine-learned force field (MLFF) phonon calculations compared to the ab-initio phonon calculations at 300 K. Visualization of data in section 4 of the SI. The different colors indicate different amounts of MD simulation steps used for training the corresponding force fields.

Figure S8: **Parity plot for vibrational energy correction ΔE_{vib} at 500 K.**

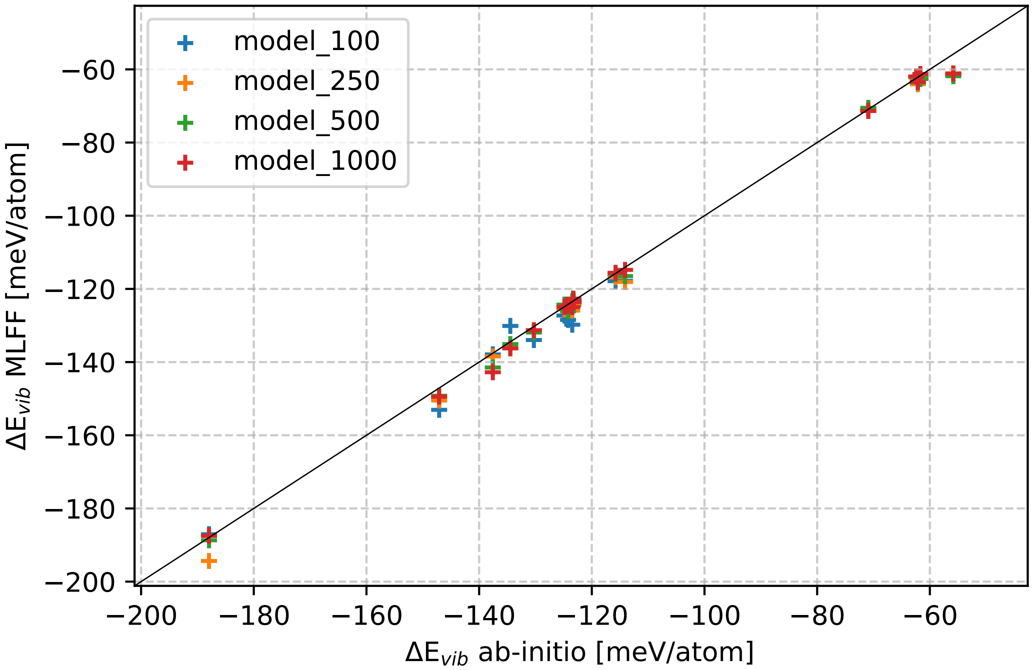

Parity plot of results for the machine-learned force field (MLFF) phonon calculations compared to the ab-initio phonon calculations at 500 K. Visualization of data in section 4 of the SI. The different colors indicate different amounts of MD simulation steps used for training the corresponding force fields.

Structural Details

Comparison $NaAlCl_4$ and $NaGaCl_4$

The following table S1 shows the high similarity between the structures of $NaAlCl_4$ and $NaGaCl_4$ regarding their lattice parameters, both for experimental structures[1] and structures obtained from a PBE-D3(BJ) optimization with a 520 eV cutoff energy.

Effect of Aliovalent Substitution on Lattice Parameters

The following table S2 shows the effect of substituting Zn^{2+} into $NaAlCl_4$ and Al^{3+} into Na_2ZnCl_4 on the lattice parameters.

Table S1: Comparison between structural properties of $NaAlCl_4$ and $NaGaCl_4$.

	Exp.[1]		PBE-D3(BJ)/520 eV	
	$NaAlCl_4$	$NaGaCl_4$	$NaAlCl_4$	$NaGaCl_4$
a/Å	9.89	9.89	9.70	9.74
b/Å	6.16	6.20	6.14	6.17
c/Å	10.32	10.32	10.12	10.13
V/Å^3	629	633	602	609

Table S2: Lattice parameters properties of substituted and unsubstituted $NaAlCl_4$ and Na_2ZnCl_4 in comparison. For comparability, the parameters of the $2 \times 1 \times 1$ supercells are compared.

ratio Zn^{2+}:Al^{3+}	a Å	Δa Å	b Å	Δb Å	c Å	Δc Å
SG 19/$NaAlCl_4$						
0:100	20.342	–	9.723	–	6.110	–
25:75	20.755	0.413	9.721	−0.002	6.163	0.053
50:50	21.660	1.228	9.862	0.139	6.045	−0.065
SG 62/Na_2ZnCl_4						
100:0	27.042	–	7.970	–	6.390	–
75:25	26.641	−0.401	7.933	−0.037	6.385	−0.005
50:50	26.248	−0.794	7.899	−0.071	6.381	−0.009

Substitution of Zn in $NaAlCl_4$

Figure S9 contains the formation energy of all calculated mixtures of Na_2ZnCl_4 and $NaAlCl_4$ from the ternary chlorides.

Figure S9: **Rescaled formation energy diagram for the mixtures of Na_2ZnCl_4 and $NaAlCl_4$.**

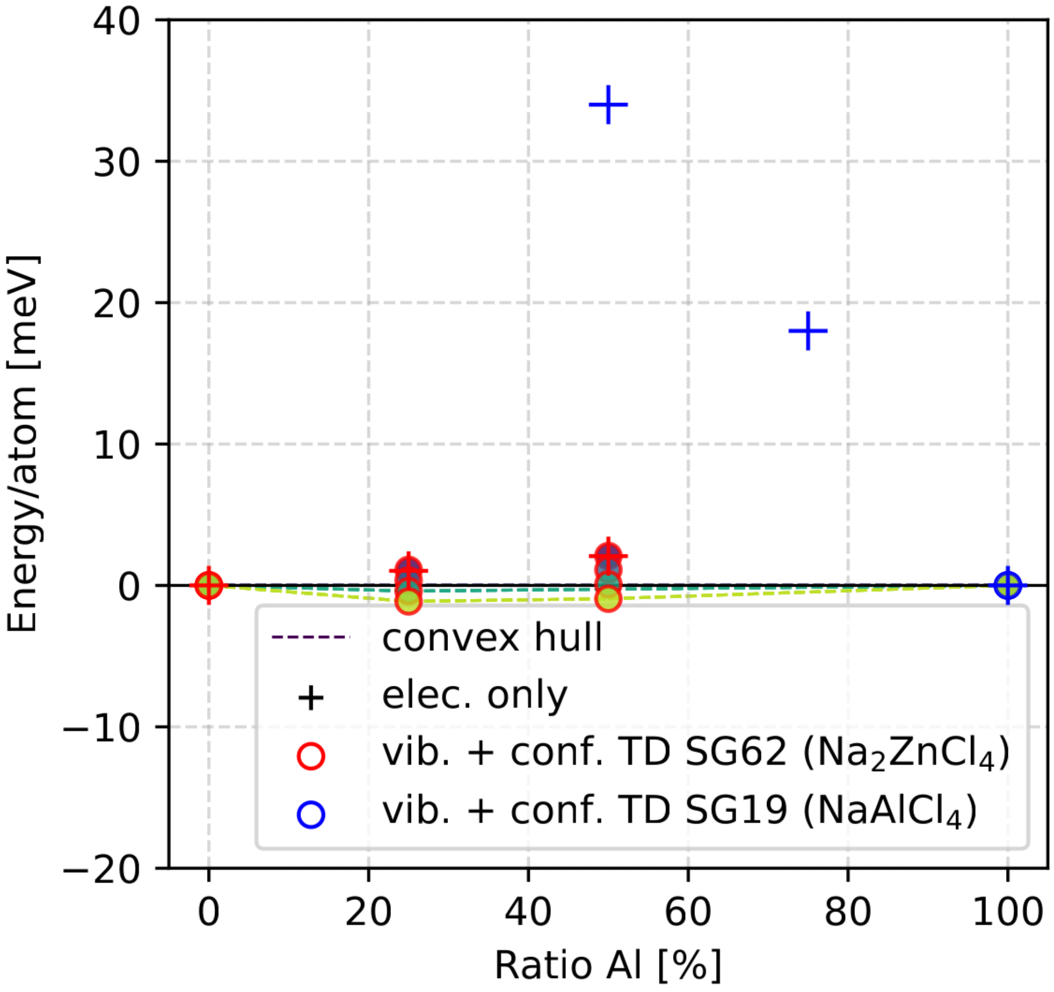

Results that contain vibrational thermodynamics and configurational entropy are indicated by circles and without thermodynamic contributions with crosses. Results for structures based on Na_2ZnCl_4 are colored red and ones base on $NaAlCl_4$ in blue. Rescaled from Figure 8 in the main part of the publication to also show the results for the mixtures based on $NaAlCl_4$.

References

(1) Meyer, G.; Schwan, E. Tetrachloroaluminate, -ferrate und -gallate: AIMIIICl4 (AI = Li-Cs, NH4, Tl, In; MIIII = Al, Fe, Ga) / Tetrachloroaluminates, -ferrates and -gallates: AIMIIICl4 (AI = Li-Cs, NH4, Tl, In; MIII = Al, Fe, Ga). *Z. Naturforsch. B* **1980**, *35*, 117–118.
